# Ecophysiological and morphological comparison of two populations of *Chlainomonas* sp. (Chlorophyta) causing red snow on ice-covered lakes in the High Tatras and Austrian Alps

**DOI:** 10.1080/09670262.2018.1426789

**Published:** 2018-04-04

**Authors:** Lenka Procházková, Daniel Remias, Andreas Holzinger, Tomáš Řezanka, Linda Nedbalová

**Affiliations:** a Charles University, Faculty of Science, Department of Ecology, Viničná 7, CZ-128 44 Prague, Czech Republic; b University of Applied Sciences Upper Austria, Stelzhamerstr. 23, A-4600 Wels, Austria; c Department of Botany, University of Innsbruck, Austria; d Institute of Microbiology of the Czech Academy of Sciences, Czech Republic

**Keywords:** Astaxanthin, *Chlainomonas*, fatty acids, field sample, High Tatras, morphology, photosynthesis, snow algae, ultrastructure, alpine lake

## Abstract

Based on analyses of multiple molecular markers (18S rDNA, ITS1, ITS2 rDNA, *rbc*L), an alga that causes red snow on the melting ice cover of a high-alpine lake in the High Tatras (Slovakia) was shown to be identical with *Chlainomonas* sp. growing in a similar habitat in the Tyrolean Alps (Austria). Both populations consisted mostly of smooth-walled quadriflagellates. They occurred in slush, and shared similar photosynthetic performances (photoinhibition above 1300 µmol photons m^–2^ s^–1^), very high levels of polyunsaturated fatty acids (PUFA, 64% and 74% respectively) and abundant astaxanthin accumulation, comparable to the red spores of *Chlamydomonas nivalis* (Bauer) Wille. Physiological differences between the Slovak and Austrian populations included higher levels of α-tocopherol and a 13Z-isomer of astaxanthin in the former. High accumulation of secondary pigments in the Slovak population probably reflected harsher environmental conditions, since the collection was made later in the growing season when cells were exposed to higher irradiance at the surface. Using a polyphasic approach, we compared *Chlainomonas* sp. with *Chlamydomonas* *nivalis*. The latter causes ʻconventionalʼ red snow, and shows high photophysiological plasticity, with high efficiency under low irradiance and no photoinhibition up to 2000 µmol photons m^–2^ s^–1^. Its PUFA content was significantly lower (50%). An annual cycle of lake-to-snow colonization by *Chlainomonas* sp. from slush layers deeper in the ice cover is proposed. Our results point to an ecologically highly specialized cryoflora species, whose global distribution is likely to be more widespread than previously assumed.

## Introduction

Red snow discolouration in alpine and polar regions is caused by many algal species (Kol, 1968; Lutz *et al*., 2015; Matsuzaki *et al*., 2015), which in most cases belong to the genera *Chlamydomonas, Chloromonas* and *Chlainomonas* (Chlorophyta) (Novis *et al*., 2008; Brown *et al*., 2016; Procházková *et al*., 2018). The genus *Chlainomonas* was established by Christen (1959), with the freshwater species *Chlainomonas ovalis* as the type. Two other species of this genus were described from snow, *C. kolii* (Hardy & Curl) Hoham and *C. rubra* (Stein & Brook) Hoham (Hoham, 1974*a*,*b*). Recently, populations of *Chlainomonas* sp. thriving periodically in slush at a high-alpine lake and at a glacier in the Austrian Alps were described (Remias *et al*., 2016). Despite morphological similarities to spherical immotile red cells of the common snow alga *Chlamydomonas nivalis*, these two Chlamydomonadacean genera are not closely phylogenetically related. Blooms of *Chlainomonas* sp. were restricted to summer snow banks with higher water content, whereas *Chlamydomonas* *nivalis* was not found in these specific habitats (Remias *et al*., 2016). However, several questions concerning the ecology and physiology of *Chlainomonas* sp. remain unanswered. For example, the photosynthetic activity in a broad range of light conditions has not yet been elucidated. Furthermore, change in fatty acid composition is one of the crucial adaptations to cold habitats (De Maayer *et al*., 2014). The fatty acid profile has not been investigated for this genus so far.

The aim of this study was to compare the ecophysiology and morphology of two populations of *Chlainomonas* sp. causing red snow on melting ice sheets in two high-alpine lakes in different European mountain ranges. We hypothesized that the population in the High Tatras (Slovakia) is the same species as the one in the Tyrolean Alps (Austria). Our intention was (1) to confirm their close phylogenetic relationship; (2) to reveal details of their cytological adaptations to the snow habitat; (3) to evaluate if the spatial distribution of the population in the ice-cover correlates with the availability of liquid water in the snow; (4) to compare the rates of photosynthesis; (5) to analyse the composition of secondary pigments; and (6) to test whether there are any differences in adaptation strategies between the populations, in terms of fatty acids. Finally, in order to evaluate differences from the snow alga *C*. *nivalis*, we investigated the same parameters and metabolites of field samples of *C. nivalis* from the Tyrolean Alps. These analyses allowed a comprehensive description of this red-snow species of the genus *Chlainomonas*, which seems to be restricted to extremophilic habitats, including slush layers in lake-ice and glacier surfaces.

## Materials and methods

### Sampling and snow characterization

**Fig. 1.**
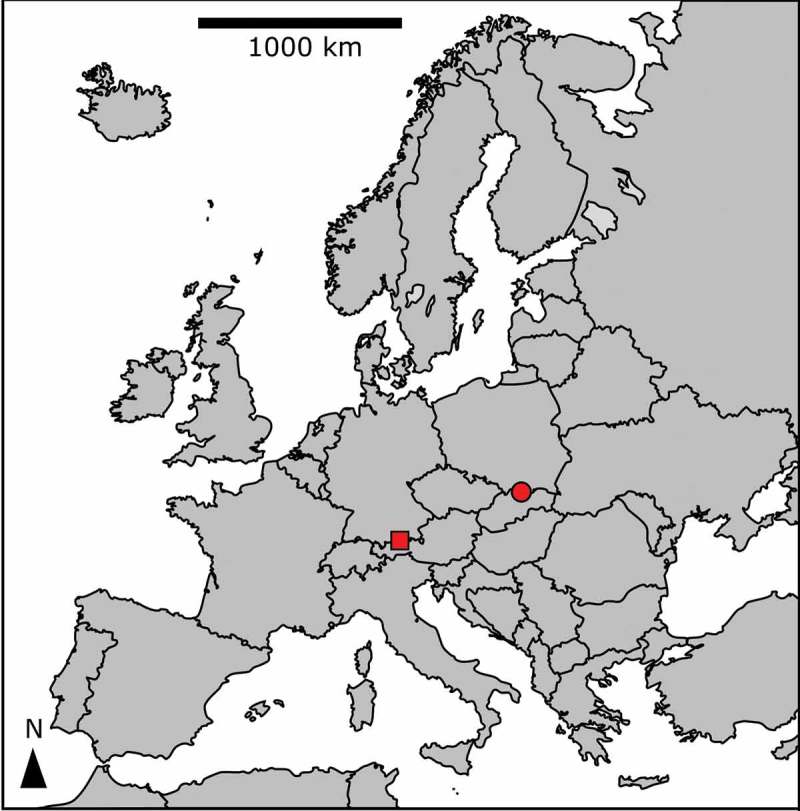
Sample locations at the High Tatras, Slovakia (circle), and the Tyrolean Alps, Austria (square).

The cryoflora causing red snow on the ice cover on Ľadové Lake (the High Tatras, Slovakia, LP03) and Gossenkӧlle Lake (Tyrolean Alps, Austria, DL06) and near Gossenkӧlle Lake (DL07) was investigated in May and June 2016 (Table 1, Fig. 1). Surface snow was harvested with a sterile shovel, placed in 10 l buckets, and transported the same day to the laboratory. Prior to photosynthesis measurements, samples were slowly melted overnight and kept in the dark at 4–5°C. Electrical conductivity (EC) and pH of the meltwater were obtained with WTW Instruments (Cond 340i and Inolab, Germany) or with HANNA (Combo EC, Romania). Snow water content (SWC) was measured by coring snow with a cylindrical polyvinylchloride corer, according to Procházková *et al*. (2018). The spatial distribution of SWC and cell densities in snow on the ice cover at Lake Gossenkӧlle were evaluated on 27 May 2016 along a transect from the southern to northern shores (Supplementary fig. S3), at the following distances from the southern shore: 1, 2, 17, 32, 47, 62, 77, 92, 107 and 108 m.

**Table 1.** Samples of *Chlainomonas* sp. from the High Tatras (Slovakia, LP03), Tyrolean Alps (Austria, DL06), and *Chlamydomonas nivalis* from the latter region (DL07) with sample codes, collection date, sampling site, altitude (m) and geographic position (GPS).

Sample	Date	Location	Altitude	GPS
LP03	19 June 2016	snow on ice cover of Ľadové Lake	2058	N49° 11.018 E20° 09.649
DL06	27 May 2016	snow on ice cover of Gossenkӧlle Lake	2411	N47° 13.762 E11° 00.885
DL07	28 May 2016	snow on slope close to Gossenkӧlle Lake	2380	N47° 13.709 E11° 00.949

### Light and electron microscopy

Light microscopy (LM, magnification 1000×) was performed and preparation of samples for scanning and transmission electron microscopy (SEM and TEM) were carried out in the same manner as described by Procházková *et al*. (2018), with the exception that from the moment of harvest, cells of the thermosensitive *Chlainomonas* sp. were immediately placed in thermos bottles to keep them cool.

### Cell counting

In order to quantify the red-snow colouration in the field, we counted the highest cell concentration. An estimation of the mean cell concentration was not intended since there were enormous differences in cell densities at the spatial scale of a dozen cm along the transect in snow at ice cover of Gossenkӧlle Lake. We took a 10 ml snow subsample from each sampling site (DL06, DL07, LP03) and from these, a further subsample of 0.5 ml snow meltwater was taken and placed in a Kolkwitz plankton-counting chamber (Hydro-Bios, Germany) and processed according to Remias *et al*. (2016).

### Isolation of DNA and sequencing

DNA was isolated as described by Procházková *et al*. (2018). The 18S small subunit ribosomal RNA gene (18S rDNA), internal transcribed spacer regions 1 and 2 (ITS1, ITS2 rDNA), and ribulose-1,5-bisphosphate carboxylase/oxygenase large subunit (*rbc*L) gene regions were amplified from DNA isolates by polymerase chain reaction (PCR), using existing primers (Supplementary table S1). Amplification and sequencing reactions for these markers were described by Procházková *et al*. (2018). New sequences were submitted to the NCBI nucleotide sequence database (accession numbers in Supplementary table S2).

### Photosynthesis

The light-dependent photosynthesis rates were obtained as the relative electron-transport rate of photosystem II with a fluorometer (PAM 2500, Heinz Walz GmbH, Germany). Cells were exposed to photon flux densities (PFDs) of 5, 34, 67, 104, 201, 366, 622, 984, 1389, 1666 and 2018 μmol photons m^–2^ s^–1^ for 30 s each at 2°C in a pre-cooled sample chamber (volume of 1 ml). Five independent biological replicates were measured for LP03, DL06 and DL07. After each light exposure, a saturating pulse was applied to detect the effective photochemical quantum yield of photosystem II. A curve of the relative electron-transport rate (rETR) upon PFD was calculated and fitted by the model according to Walsby (1997), assuming photoinhibition. Relative and maximum electro-transport rate (ETR_max_), initial slope (α) and light saturation point (I_k_) were determined (Procházková *et al*., 2018).

### Pigment analysis

Chlorophylls, carotenoids and tocopherols were extracted and quantified according to Remias & Lütz (2007). Briefly, cells were freeze-dried on glass-fibre filters, disrupted with a grinding mill and extracted with dimethylformamide. The analysis was performed with an Agilent 1100 HPLC system with a LiChroSpher C18 column and diode array and fluorescence detectors. Carotenoid standards were obtained from CarotNature (Switzerland).

### Lipid extraction and analysis of fatty acid methyl esters (FAMEs)

The extraction procedure was based on the method of Bligh & Dyer (1959), and elution was done from a Sep-Pak Vac Silica cartridge 35cc (Waters; 10 g normal-phase silica) by chloroform (neutral lipids), acetone (glycolipids) and methanol (phospholipids) (Saunders & Horrocks, 1984). All classes of lipids were saponified overnight in 10% KOH in methanol at room temperature. The structures of FAMEs were confirmed by comparison with GC/MS retention times, and fragmentation patterns with those of standard FAMEs (Supelco, Prague) (Řezanka, 1990; Dembitsky *et al*., 1991). Procedures were described in detail by Procházková *et al*. (2018).

### Climatic conditions

For a comparison of the prevailing climate above the snow surface at the sampling localities in the two mountain ranges in the course of a year, radiation, monthly and daily mean air temperature (°C), and monthly cumulative precipitation (mm) were used. For complete description see the Supplementary text.

## Results

### Collection sites and habitat conditions

**Figs 2–5.**
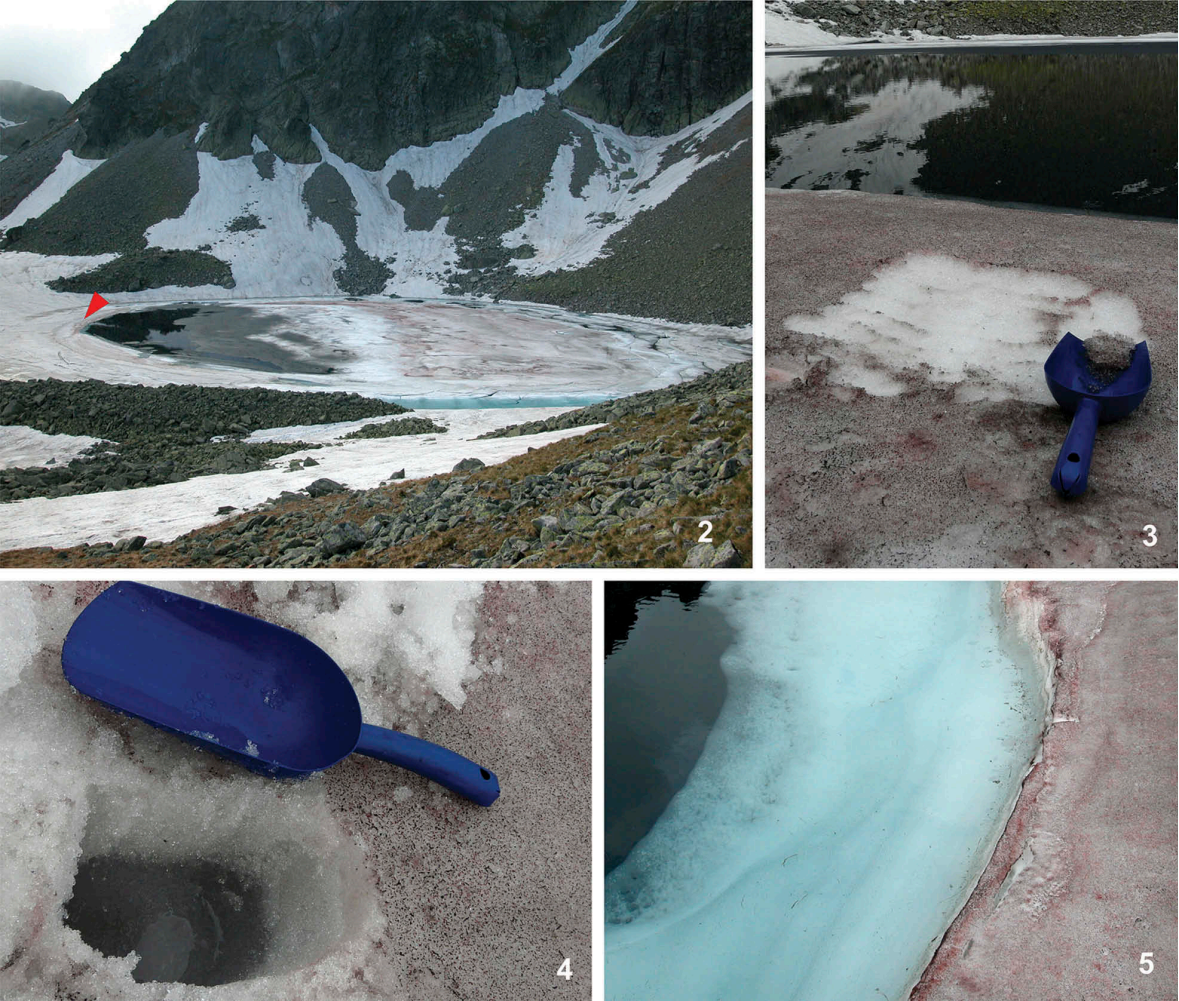
Overview of the sampling site of *Chlainomonas* sp. at Ľadové Lake (the High Tatras, Slovakia). **Fig. 2**. Red colouration was visible at nearly all ice-covered parts of the lake (mid-June 2016). The harvest spot was close to the southern shore (sample LP03, red arrowhead). **Fig. 3**. Detailed view of red snow after harvest, *Chlainomonas* sp. was present at the surface and down to a depth of 2 cm. **Fig. 4**. Approximately 10 cm below the surface a prominent soft slush layer was noticed, which was apparently soaked by lake water. **Fig. 5**. Detail view of the interface between the lake margin with red snow close to the shore and lake water with edges of a slush layer.

Red snow caused by *Chlainomonas* sp. was found in late spring 2016 on two still partly ice-covered high-alpine lakes, Ľadové Lake in the High Tatras and Gossenkӧlle Lake in the Tyrolean Alps (Table 1). In the High Tatras, the lake was partly ice-free and red snow was visible on all remaining ice-covered parts (Figs 2–5). The texture of this snow was partly slushy and partly frozen (sample LP03). A prominent soft slush layer, which was apparently soaked by lake water, began approximately 10 cm below the surface. At the Tyrolean location, the snow colouration was visible only close to the lake shore, where melting of the underlying ice was advanced (sample DL06; Supplementary figs S1–S4). The majority of the lake surface was still covered with white snow, and red horizontal patches of snow populated by *Chlainomonas* sp. were hidden several centimetres below the snow surface, close to the interface with the ice cover. One week later, red spots appeared across the entire lake on the surface of the snow. For a comparison ‘terrestrial’ red snow caused by *C*. *nivalis* was collected near Gossenkӧlle Lake (sample DL07). This sample was dominated by blood-red mature spores, with a smaller contribution (15%) of young cells in an early stage of development, with green parts of the chloroplasts visible. The habitat conditions of all localities are summarized in Table 2.

**Table 2.** Abiotic habitat parameters and cell sizes of snow algae in field samples from the High Tatras (LP03) and Tyrolean Alps (DL06, DL07).

Species (sample)	EC	pH	SWC	Population density ml^–1^	Cell length	Cell width
*Chlainomonas* sp. (LP03)	n.a.	5.8	56.4±3.8	44150±3091	37.7±7.5	35.9±7.3
*Chlainomonas* sp. (DL06)	2.5	5.8	57.9±1.6	6728±619	30.7±6.2	29±5.9
*Chlamydomonas nivalis* (DL07)	8.4	6.2	53.9±2.4	56036±3867	16.3±4.2	15.7±4.3

Electrical conductivity (EC; μS cm^–1^), pH of meltwater and snow water content (SWC; %), population density (cells ml^–1^ meltwater) ± SD (standard deviation), mean sizes of cells in μm ± SD, n.a. – not available.

### Morphology and ultrastructure

**Figs 6–14.**
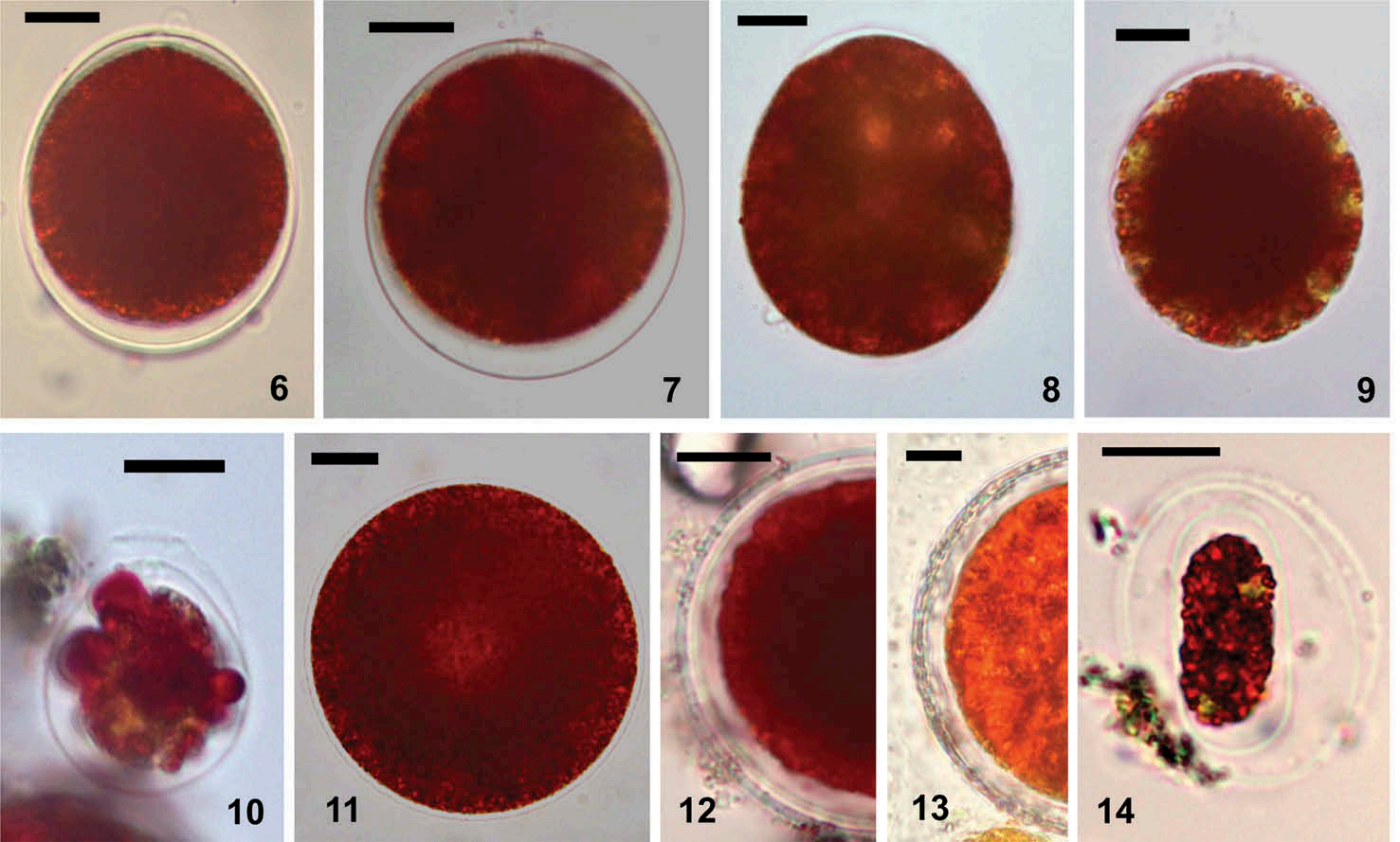
LM micrographs of *Chlainomonas* sp. showing cells from snow at the ice cover of the Ľadové Lake directly after harvest (Figs 6–11) and after several months at lab conditions (Figs 12–14). **Figs 6–10**. Morphological variability of swarmers. **Fig. 6.** Typical swarmer possessed papilla and pseudo-papilla (suggested zygote). **Fig. 7.** Rarely, the cell wall was more distant from the protoplast all around. **Figs 8, 9.** Motile cells with a single thin cell wall and a papilla. Note greenish spots of the parietal chloroplasts. **Fig. 10.** A flagellate with a collared papilla. **Fig. 11.** Non-motile stage without partially thickened cell wall, note central brighter region most likely representing the position of the nucleus. **Figs 12, 13**. Mature spores. **Fig. 12.** Smaller spore with hyaline primary cell wall and multiple layers of secondary cell walls. **Fig. 13.** During ageing of spores, the secondary cell wall becomes thicker. **Fig. 14**. Oblong flagellate containing red pigments and a few greenish spots of parietal chloroplasts. It is probably a daughter cell still remaining in the spore. Scale = 10 µm.

The morphology of *Chlainomonas* sp. was characterized by LM (Figs 6–14 for the High Tatras and Supplementary figs S5–S12 for the Tyrolean Alps). The populations at the two locations had very similar morphology. Swarmers of *Chlainomonas* sp. had four flagella. Each flagellum was about as long as the cell (Table 2). The ellipsoidal to nearly spherical flagellates were morphologically variable: many of them possessed a papilla (thickened cell walls with flagellar openings at the anterior pole) and a pseudo-papilla (thickened posterior cell wall) (Fig. 6, Supplementary fig. S5); rarely, the cell wall was entirely detached from the protoplast (Fig. 7). Additionally, swarmers with an equally thin cell wall and papilla (Figs 8, 9, Supplementary figs S6, S7) and swarmers with a collar-like papilla (Fig. 10, Supplementary figs S8–S11) occurred. The protoplast was almost entirely occupied with red pigment. In a few cases, greenish spots of a parietal chloroplast were visible (Fig. 9).

**Figs 15–18.**
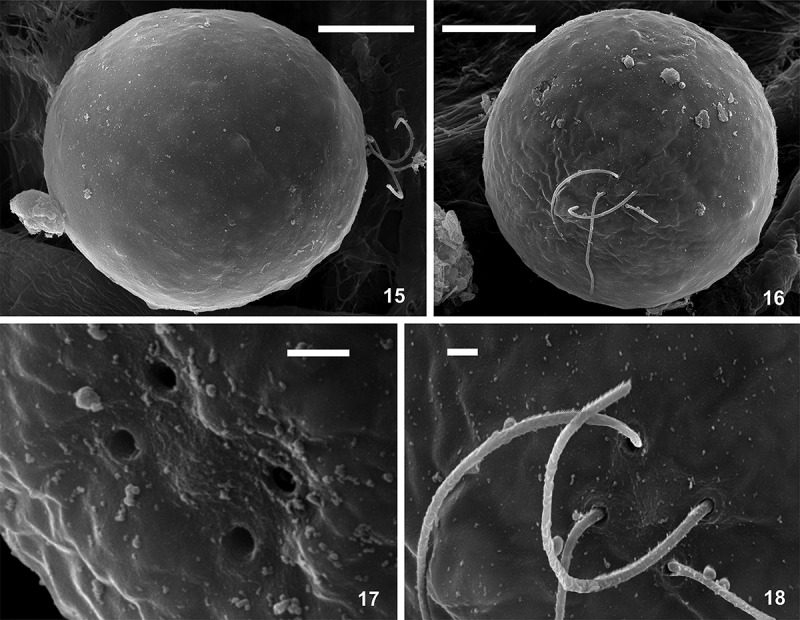
SEM micrographs of *Chlainomonas* sp. swarmers from the snow of the Ľadové Lake. **Figs 15, 16**. Side and apical view showing the smooth surface of quadriflagellate cells. **Fig. 17**. Detail view of four spherical flagellar grooves in slightly rectangular position. **Fig. 18**. Detail view of two pairs of flagella. Scale = 10 µm (**Figs 15**, **16**) and 1 µm (**Figs 17**, **18**).

The mean cell sizes of the most common, non-collared flagellates of *Chlainomonas* sp. from both locations are summarized in Table 2. Other life-cycle stages occurred rarely (<5%), and their cell sizes are shown in Supplementary table S3. Flagellates with collared papillae were smaller than the dominant non-collared flagellates. Immotile stages were ovoid to spherical (Fig. 11). Maintaining field-collected material in melt-water at 4°C in the laboratory for several months helped to reveal other life-cycle stages, which were rarely found in the field. First, mature spores developed from quadriflagellate swarmers, their primary cell wall became hyaline, and a new secondary cell wall was formed below. Sometimes, a third cell wall or multiple layers were also present (Figs 12, 13, Supplementary fig. S12). Second, smaller oblong red biflagellates enclosed by two layers of a mother cell wall (likely derived from mature spores) appeared in a subsample kept in the same conditions but in the dark (Fig. 14). Further cellular details such as the stigma, cell-wall surface structures such as spines, cell divisions, or other putative stages in the life cycle were not observed. The cell-wall surface of *Chlainomonas* sp. was depicted by SEM (Figs 15–18). A smooth surface was characteristic for swarmers (Figs 15, 16). Four spherical flagellar grooves in a subrectangular arrangement were found (Figs 17, 18). Mature spores of *Chlainomonas* sp. possessed fine structures arranged in such a way as to lend an undulating appearance (Supplementary fig. S15, corresponding to Supplementary fig. S12).

**Figs 19–24.**
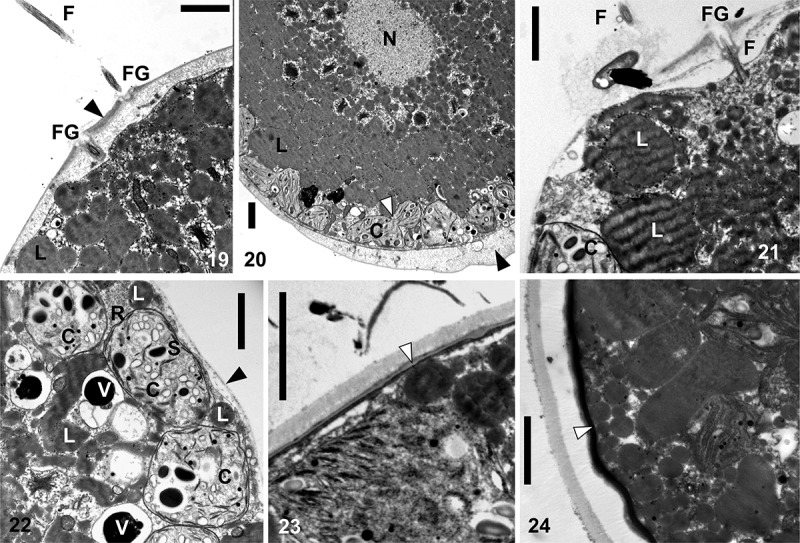
TEM micrographs of *Chlainomonas* sp. swarmers (Figs 19–22) and spores (Figs 23, 24) from the snow of the Ľadové Lake. **Figs 19, 20**. A section showing flagellate (F) and two flagella grooves (FG) of a swarmer. Cell wall thickened at the anterior and the posterior of the cell (black arrows). Note small chloroplasts (C) located parietally and a putative process of plastid division (white arrow). Centrally located nucleus (N), likely surrounded by many lipid bodies (L). **Figs 21, 22**. A swarmer with a single, equally thin cell wall (black arrow) with papilla. Section showing one flagella (F) and the flagella groove (FG). Note lipid bodies (L), starch grains (S) in chloroplasts (C), electron dense vacuoles (V) containing a crystalline content and a ribosome rich region (R) close to the cell wall. **Figs 23, 24**. Spherical spore with the trilaminar sheath (secondary cell wall, white arrow). Later outer layers of extracellular matrix are developed. Scale = 2 µm.

The ultrastructure of *Chlainomonas* sp. was analysed by TEM (Figs 19–24 for LP03; Supplementary figs S14, S16, S17 for DL06). The cell walls of most swarmers were thickened at the anterior and posterior ends (Figs 19, 20). A section showing two flagella is depicted in Fig. 19. Small plastids containing starch grains were located parietally (Figs 20, 22). No pyrenoid was observed. The nucleus was positioned centrally and surrounded by many lipid bodies (Fig. 20). Swarmers with a single, uniformly thin cell wall were also found (Figs 8, 9, 21, 22; Supplementary figs S6, S7, S14). Mature spores possessed a trilaminar sheath (secondary wall) surrounded by outer layer(s) of extracellular matrix (Figs 23, 24, Supplementary figs S16, S17). All observed cell stages contained cytoplasmic electron-dense vacuoles, commonly filled with crystalline structures (Fig. 22).

### Population density

The population densities of *Chlainomonas* sp. were 6728±619 and 44150±3091 cells ml^–1^ melt-water in the samples from the Tyrolean Alps and the High Tatras, respectively (Table 2). Population densities and SWC were investigated in a 109 m-long south-north transect on the ice cover of Lake Gossenkӧlle (Supplementary figs S18, S19). The highest values of SWC were reached at sampling points closest to both lake shores (89.3±1.5% and 80.8±2.9%). At points more distant from the lake shores, SWC was significantly lower and ranged from 50.5±2.6 to 57.9±1.6% (Supplementary fig. S19). The cells were least abundant in the central part of the transect (<400 cells ml^–1^ meltwater), and most abundant in the slushy part, in close proximity to the northern lake shore (>10 000 cells ml^–1^ meltwater) (Supplementary fig. S18). In contrast, the slushy area next to the southern lake shore harboured only one-third as many cells per volume. The spatial variation at the other sampling points of the transect was considerable, ranging from >1500 to <7500 cells ml^–1^ meltwater.

### Snow-algal identity inferred from molecular markers

Analyses of the molecular markers 18S rDNA, ITS1 rDNA, ITS2 rDNA and *rbc*L showed that the red snow of the ice covers of both lakes (samples LP03 and DL06) was caused by the same species (100% identity between markers of the two populations). Furthermore, 18S rDNA and *rbc*L sequences were identical to those of *Chlainomonas* sp. in previous reports (GU117574.1, LN897303; Remias *et al*., 2010, 2016). The 18S rDNA and ITS2 rDNA for *C*. *nivalis* (DL07) causing red snow on slopes neighbouring Lake Gossenkӧlle was, with the exception of one nucleotide change at ITS2 rDNA, identical to a field sample found at a location 40 km south-west (GU117577.1, Remias *et al*., 2010).

### Photosynthesis

**Fig. 25.**
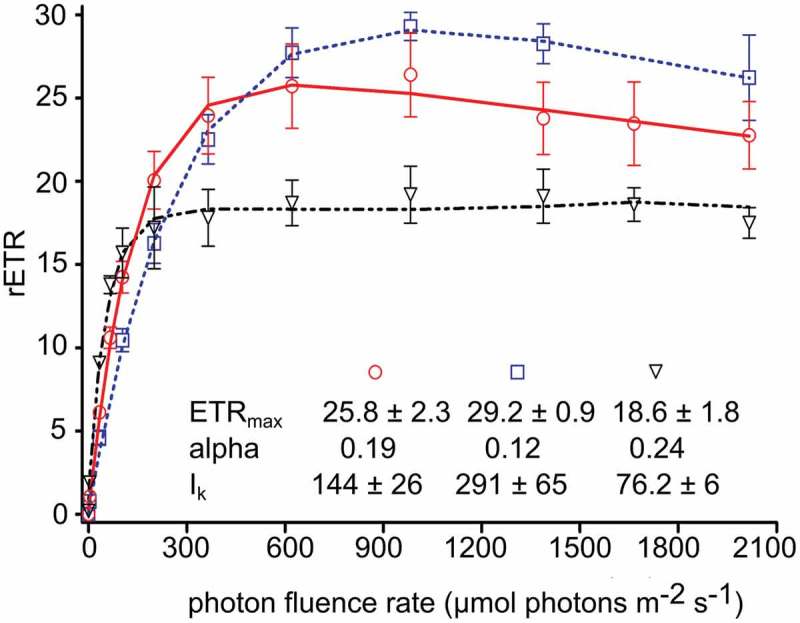
Comparison of the light-dependent relative electron transport rate (rETR) between two genera causing red snow: *Chlainomonas* sp. swarmers inhabiting ice-covered high alpine lakes (two samples: LP03 (High Tatras) – circles, DL06 (Tyrol Alps) – squares) and *Chlamydomonas nivalis* spores thriving in terrestrial snow habitats (DL07 – triangles). Values of maximum relative electron transfer rate (rETR_max_), initial slope (α) and saturation irradiance (I_k_) for both genera are shown. The data points were fitted with the model of photoinhibition according to Walsby model (1997) assuming photoinhibition. Each symbol represents the mean value of five replicate measurements (± SD).

*Chlainomonas* sp. from the High Tatras showed an α value of 0.19±0.02, a relative ETR_max_ of 25.8±2.3 and an I_k_ value of 144±26 μmol photons m^–2^ s^–1^ (Fig. 25). *Chlainomonas* sp. from the Tyrolean Alps showed a similar photosynthetic performance (Fig. 25). In both locations, photoinhibition occurred above 1300 μmol photons m^–2^ s^–1^. The only significant differences were a one-third lower α value (0.12±0.01), a slightly higher ETR_max_ (29.2±0.9), and a two-fold higher I_k_ value (291±65 μmol photons m^–2^ s^–1^) for the latter population. In contrast, *C*. *nivalis* showed signs of photoinhibition beginning only at much higher irradiances (2000 μmol photons m^–2^ s^–1^); it also showed a lower ETR_max_ (18.6±1.8) and I_k_ (76.2±6), but a higher α (0.24).

### Pigment composition

The reddish colouration of *Chlainomonas* sp. in the High Tatras and the Austrian Alps was caused by secondary (non-plastidal) carotenoids, which comprised 93% and 88.5% of all pigments, respectively. These pigments were identified as derivatives (likely esters) of the keto-carotenoid astaxanthin (Supplementary figs S20, S21). Chlorophyll-*a* and -*b* comprised 5% and 8% of all pigments, primary (plastidal) carotenoids represented 2% and 3.5% in the pigment pool. For astaxanthin, 44.5% (High Tatras) and 31.9% (Alps) occurred as the native 13Z isomer. The overall ratio of astaxanthin to chl-*a* was 26 and 17 to one, respectively. Other pigment contents are given in Supplementary table S4.

### Fatty acid composition

The relative contents of FAs (as % of total lipids and as % of the three major lipid groups) in three snow-algal field samples are shown in Table 3. The Slovak and Austrian populations of *Chlainomonas* sp. had very high levels of polyunsaturated fatty acids (PUFAs; 64.4% and 74.2% of total lipids), whereas the content of saturated acids (SAFAs) did not exceed 27% or 21% (mainly palmitic acid, 16:0 and stearic acid, 18:0), respectively. The contribution of monounsaturated fatty acids (MUFAs) was low (<10% of total lipids), with oleic acid (18:1 (9Z)) the most abundant. The major PUFAs were α-linolenic acid (18:3 (9Z,12Z,15Z)), followed by stearidonic acid (18:4 (6Z, 9Z,12Z,15Z)) and linoleic acid (18:2 (9Z,12Z)). For comparison, *C*. *nivalis* had significantly lower levels of PUFAs (50% of total lipids) (Table 3). The content of SAFAs did not exceed 30%, resulting in a two-fold higher contribution of MUFAs (20% of total lipids) in comparison with *Chlainomonas* sp. Apart from oleic acid, vaccenic acid (18:1 (11Z)) was the second most abundant MUFA. The major PUFAs for *C*. *nivalis* were the same as for *Chlainomonas* sp. Composition of three major lipid groups differed in saturation of their fatty acids: neutral lipids were composed predominantly of saturated lipids, whereas phospholipids and glycolipids were composed predominantly of PUFAs (Table 3). The total lipid contents of the dry biomass were about 10% for both *Chlainomonas* sp. populations and for *C*. *nivalis* (Table 3). *Chlamydomonas nivalis* differed in the PUFA profile of its biomembranes (phospholipids and glycolipids) from those of *Chlainomonas* sp. in the nearly 10-fold higher and 3-fold lower contents of vaccenic acid and linoleic acid in *Chlamydomonas* *nivalis*.

**Table 3.** Snow algal fatty acid composition in % of total lipids (TL) and in % of the three major lipid groups: neutral lipids (NL), phospholipids (PL) and glycolipids (GL).

	*Chlainomonas* sp. LP03				*Chlainomonas* sp. DL06				*Chlamydomonas nivalis* DL07			
	TL	NL	PL	GL	TL	NL	PL	GL	TL	NL	PL	GL
14:0	3.0	5.0	0.9	3.0	1.5	3.9	0.8	0.8	0.7	0.9	0.6	0.5
16:0	19.1	37.8	13.2	7.9	13.3	37.4	4.7	3.5	19.6	53.0	7.8	6.3
16:1 (9Z)	0.7	0.4	0.9	0.9	0.9	0.8	0.9	0.9	1.2	1.5	1.0	0.9
16:1 (11Z)	0.1	0.0	0.4	0.2	0.2	0.2	0.3	0.3	1.3	1.6	1.1	1.0
16:2 (7Z,10Z)	2.0	1.0	2.3	2.3	1.8	0.9	2.1	1.7	1.6	2.0	1.4	1.3
16:3 (4Z, 7Z,10Z)	3.0	2.0	3.4	3.7	3.4	2.0	3.8	4.0	2.1	2.7	1.8	1.6
16:3 (7Z, 10Z, 13Z)	1.4	1.0	2.5	2.2	1.8	1.0	2.7	2.0	0.9	0.4	0.9	1.1
16:4 (4Z, 7Z, 11Z, 13Z)	3.5	2.0	4.2	5.4	4.2	3.0	4.7	4.1	5.6	3.8	6.0	4.7
18:0	4.2	1.0	5.5	6.6	5.7	2.0	10.4	8.5	9.7	2.5	12.1	7.9
18:1 (9Z)	8.1	13.9	3.8	4.9	3.4	6.9	3.8	4.2	9.1	10.1	9.5	9.4
18:1 (11Z)	0.4	1.0	0.7	0.7	0.8	0.6	1.4	1.2	8.3	7.6	11.2	11.8
18:2 (9Z, 12Z)	14.1	12.0	15.1	14.8	11.6	8.9	12.3	14.4	4.3	2.5	4.8	4.7
18:3 (9Z, 12Z, 15Z)	22.2	14.9	26.4	26.7	32.9	18.7	32.2	35.6	26.4	10.1	27.6	32.2
18:4 (6Z, 9Z, 12Z 15Z)	18.2	8.0	20.7	20.7	18.5	13.7	19.9	18.8	9.2	1.3	14.2	16.6
SUFA	26.3	43.8	19.6	17.5	20.5	43.3	15.9	12.8	30.0	56.4	20.5	14.7
MUFA	9.3	15.3	5.8	6.7	5.3	8.5	6.4	6.6	19.9	20.8	22.8	23.1
PUFA	64.4	40.9	74.6	75.8	74.2	48.2	77.7	80.6	50.1	22.8	56.7	62.2

The table shows only fatty acids that have abundances greater than 0.1%. The relative proportion of saturated (SAFA), monounsaturated (MUFA) and polyunsaturated (PUFA) fatty acids is also given.

### Climatic conditions

The air temperature in close proximity to Ľadové Lake and Gossenkӧlle Lake followed the same pattern over the course of a year (Supplementary fig. S23). The precipitation regime in late spring and early summer differed (Supplementary fig. S24).

## Discussion

### Taxonomy and related species

Melting ice layers of both high-alpine lakes were populated by the same species. *Chlainomonas* sp. groups among the two other cryoflora species of this genus (see the phylogenetic tree by Remias *et al*., 2016 in fig. 3). According to their *rbc*L phylogeny, the populations in this study belong to a lineage independent from any other known *Chlainomonas* species (Remias *et al*., 2016), although their cell size ranges overlap (Supplementary table S3). In order to determine their affiliation, a multigene analysis will be necessary, which is not yet possible because no molecular markers (except *rbc*L) are available for other *Chlainomonas* species. In addition, the sole use of the *rbc*L gene in the phylogeny of *Chloromonadinia* is problematic because unusual gene substitutions, which occur frequently in this group, may result in misleading taxonomic artefacts (Nozaki *et al*., 2002, 2010). *Chlainomonas* sp. seems to be in close affinity (99% identity at 18S rDNA) to two algae tentatively assigned as ʻ*Chloromonas* sp. TA1ʼ (AB903004.1) and ʻ*Chloromonas* sp. TA3ʼ (AB902981.1), which were isolated from red snow at alpine sites in Japan, at an elevation of 2270 m. However, *Chlainomonas* sp. from Slovakia and Austria represents an independent species from these Japanese snow-algal samples (only 69% similarity at ITS2 rDNA, AB903004.1 and AB902981.1). We refrain from generating a new phylogenetic tree, because for all molecular markers investigated, no new molecular information has become available since the study by Remias *et al*. (2016).

### Cytological adaptations to the snow habitat

At both locations, the dominant stages were non-collared ovoid swarmers with four flagella and abundant red pigmentation. Occurrence of swarmers with collar-like papillae (corresponding to the life-cycle stage shown by Novis, 2001, 2002*b*) would morphologically point to a traditional designation as *Chlainomonas kolii*. However, the proportion of collar-like flagellates in populations is probably a dynamic process (Novis, 2002*a*). Thus, the taxonomic value of this feature is subject to discussion. Additionally, cell sizes of dark-red oblong biflagellate swarmers corresponded to the morphotype found in populations of both *C*. *kolii* in New Zealand (Novis, 2002*b*) and *C. rubra* in North America (Hoham, 1974*a*). Very probably, these small oblong biflagellates are daughter cells released from spores after germination, as shown by Hoham (1974*a*) for *C*. *kolii*. We hypothesize that the smaller elongate biflagellates represent the vegetative stage, whereas the quadriflagellate swarmers are prolonged planozygotes (as considered by Stein & Brooke, 1964; Hoham, 1974*a*), which later become spherical and immotile zygotes. The quadriflagellate swarmers of *Chlainomonas* sp. observed here share the arrangement of unequal flagella insertion as described for *C*. *kolii* (Novis *et al*., 2008), where the flagellar basal apparatus was organized as two distinct pairs of basal bodies that lack any significant connections. This observation supports the speculation that these stages represent planozygotes originated from a fusion of two biflagellate swarmers. Consequently, the genus *Chlainomonas* might be invalid, in view of the finding that molecular markers place it within the genus *Chloromonas* (Novis *et al*., 2008), where vegetative swarmers are generally biflagellate. These presumed planozygotes described in this study probably do not have well-developed mechanisms of mechanical resistance or thermostability similar to the mature immotile stages. Thus, their protoplast is quickly damaged after being frozen below 0°C (Hoham, 1975; this study) or when suffering heat stress (Remias *et al*., 2016; this study). The process of plastid reorganization of *Chloromonas* spp. in the course of their life cycle (Remias *et al*., 2010; Procházková *et al*., 2018) seems to be valid also for *Chlainomonas*, as occasionally we found thin-walled swarmers with one or a few larger plastids close to the central part of a cell. The ‘final seasonal stage’ morphotype for *Chlainomonas* sp. had a reticulate surface, which is common for some other members of Chlamydomonadaceae (VanWinkle-Swift & Rickoll, 1997; Malmberg & VanWinkle-Swift, 2001). The thick-walled mature spores of *Chlainomonas* sp. seem to be adapted to survive harsh conditions, including starvation, mechanical abrasion, freezing and desiccation (Holzinger *et al*., 2016). Cytokinesis, as reported by Hoham (1974*b*) and Novis *et al*. (2008), was not noted in this study. As sexual reproduction was not directly observed and attempts to generate a strain were unsuccessful, further details of the life cycle of *Chlainomonas* sp. remain unknown.

### Spatial distribution and habitat conditions

The blooms of *Chlainomonas* sp. seem to be an annual phenomenon at Ľadové Lake (tentatively identified as ʻ*Chlamydomonas* cf. *nivalis*ʼ by Nedbalová *et al*., 2006) and at Gossenkӧlle Lake (Remias *et al*., 2016) – lakes which share several characteristics of morphometry and limnochemistry (Kamenik *et al*., 2000; Kopáček *et al*., 2006). Moreover, both lakes are covered with ice and snow for more than seven months per year (Felip *et al*., 2002; Šporka *et al*., 2006), and the ice cover reaches a maximum thickness of 2.5 m (former) or up to one-third of the lake volume (latter). Red snow has never been reported from neighbouring lakes in the same valleys, probably as a result of their geomorphological settings: shallow lakes with rather flat beds usually have less-developed ice cover and associated snowpacks (Šporka *et al*., 2006). In the two lakes inhabited by *Chlainomonas*, high snow accumulations turn ice covers into complex structures consisting of several ice layers (Sattler *et al*., 2012). Highly active microbial communities in slush layers of the winter cover (Felip *et al*., 1999) can be colonizers of the lake water column (Alfreider *et al*., 1996) and vice versa (Felip *et al*., 1995). Thus, one may suggest that the *Chlainomonas* population causing red snow on melting lake ice covers is derived from lake slush layers – the algae are released into the water column after complete snowmelt and enter the phytoplankton (Nedbalová *et al*., 2006). In fact, several large-sized red-pigmented volvocalean cells (tentatively identified as ʻ*Chlamydomonas* sp.ʼ, ʻ*Pteromonas*ʼ and ʻ*Chlamydomonas nivalis*ʼ) have been found in the slush layers and small pools on the top of the ice cover of Gossenkölle Lake (Felip *et al*., 2002). In this lake, some of these cell morphotypes most likely represent stages in the life cycle of *Chlainomonas*, as proposed by Novis (2001). Comparison of mean population abundances in lake snow and at the lake shore, the latter being significantly lower (>1000 cells ml^–1^ vs. <50 cells ml^–1^, Novis, 2002*a*), support a concept of annual colonization of the lake ice-cover by *Chlainomonas* sp. mainly from the water column rather than from contributing surface streams. A patchy distribution of *Chlainomonas* sp. along the south-north transect observed in this study (Supplementary fig. S18) corresponds to a considerable spatial variation in population densities within the algal bloom, as also shown for *C*. *kolii* in New Zealand (Novis, 2002*a*). A population from the Austrian Alps reached, in the course of our study, comparable densities to previous years (Remias *et al*., 2016). However, this population was sparse in comparison with the four-fold higher abundances at Ľadové Lake, which can be attributed partly to the collection later in the season. Generally *Chlainomonas* sp. was found in slush or nearly completely melted snow on the lake ice cover, but it has also been reported from a glacier in Austria (Remias *et al*., 2016). In a similar way, *C. kolii* caused snow discolouration in a slush layer (SWC 69%) over snow with a lower density (SWC 46%) (Hardy & Curl, 1968). In our field samples, no cells in the process of division were found. Despite very rare observations of sporangia reported for *C*. *kolii* (Novis, 2002*a*), population densities can show significant passive increases, associated partly or even solely with ablation of snow (Novis, 2002*a*), which can explain the absence of any correlation between the SWC and population densities in this study. Taking these possibilities into account, we assume that cell division and gamete mating are most likely already taking place in the slush layers prior to their exposure to the surface. This generally corresponds to the finding that sexual stages of *Chloromonas* snow algae occur if the SWC is lower (39–50%, L Procházková, unpublished observation; 47–54% observed by Hoham & Duval, 2001).

### Morphologically similar algae and their habitat preferences

Three species or subspecies of red snow-causing algae are known from the High Tatras, namely *Chlamydomonas nivalis* (Kol, 1975*a*, 1975*b*), *Chloromonas nivalis* subsp. *tatrae* (Kol) Procházková, Remias, Nedbalová & Řezanka (Procházková *et al*., 2018), and *Chlamydomonas sanguinea* Lagerheim (Kol, 1969). According to Remias *et al*. (2016) this last, poorly morphologically described, species looks very similar to *Chlainomonas* sp. *Chlamydomonas sanguinea* occurred on the Slovak side (Kol, 1975*a*, 1975*b*) and probably also on the Polish side of the High Tatras (misidentified as *Chlamydomonas nivalis*; Kawecka, 1981). In the Tyrolean Alps, the first report of *Chlainomonas* was from the small glacier Gamezkogelferner, not far from Gossenkӧlle Lake (Ettl, 1968), and corresponded to scattered cells found in the course of this study in snow (Supplementary fig. S13). The original description of *Chlainomonas rubra* included cells with striking spikes on the wall surface (Stein & Brooke, 1964). The second *Chlainomonas* species living in snow, *C*. *kolii*, differs from *C*. *rubra* in the presence of swarmers with a collar-like papilla with an outer, ephemeral cell wall consisting of mosaic plates. Similar to our observation of the habitat preference for *Chlainomonas* sp. in Europe, remarkable blooms of the related *C*. *kolii* were recorded in the snow of the ice covers of an alpine lake on Mt Philistine and on Canyon Lake, New Zealand (Novis, 2002*a*, 2002*b*; Novis *et al*., 2008), and *C*. *rubra* in the similar habitats of Squaw Lake and Upper Lena Lake, United States of America (Novis *et al*., 2008). Therefore, a worldwide distribution of the genus *Chlainomonas* in snowpacks associated with high-alpine lacustrine ecosystems seems to be more common than previously thought. In addition, *C*. *rubra* is known from snowpacks above the timberline on the volcanic Mt Ruapehu in New Zealand (Hardy, 1966), and from the Vitosha Mountains in Bulgaria (fig. 39 in Lukavský *et al*., 2009). According to other North American reports, *C*. *rubra* and *C*. *kolii* were found beneath or adjacent to coniferous canopies (Stein & Brooke, 1964; Hardy & Curl, 1968; Hoham, 1974*a*, 1974*b*).

### Photosynthesis

The photosynthetic rates of *Chlainomonas* sp. from both sites were consistent in their relationship to irradiance and in suffering photoinhibition at very high light intensities, from 1300 µmol photons m^–2^ s^–1^ upwards, which are common at open, high-alpine sites. *Chlainomonas* sp. (DL06) appears to tolerate higher light intensities, where it shows very high photosynthetic performance. Under low light, photosynthesis is less efficient; however, both show similar kinetics. In contrast, the ‘terrestrial’ snow alga *C*. *nivalis* behaves differently, showing high photophysiological plasticity: high photosynthetic efficiency under low light, but less photoinhibition under high light. As indicated by the I_k_, *Chlainomonas* sp. from the High Tatras needs only half the level of irradiance compared with the Tyrolean population to become saturated. This can be explained by the pronounced temperature stress and associated irradiance stress experienced later in the season for the population sampled at Ľadové Lake (Supplementary figs S25, S26), in comparison to the population sampled earlier at Gossenkölle Lake. An increased accumulation of *Chlainomonas* sp. at the snow surface is expected as a consequence of rain events (Novis, 2002*a*), which are more frequent in the High Tatras (Niedzwiedz, 1992). The pronounced topographic shading of Ľadové Lake may also contribute to the observed photophysiological differences (Supplementary figs S27, S28; Novikmec *et al*., 2013), although maximum irradiance levels during the day can reach comparable levels of 2500 µmol photons m^–2^ s^–1^ (Sommaruga & Psenner, 1997; Procházková *et al*., 2018).

### Pigments

The high levels of astaxanthin causing the red colouration of *Chlainomonas* sp. are comparable to mature *C*. *nivalis* obtained from alpine (Bidigare *et al*., 1993; Remias *et al*., 2005) and polar regions (Müller *et al*., 1998). The higher astaxanthin: chlorophyll ratios for the High Tatras population than for the earlier-sampled Austrian Alps population may be a result of the ongoing season, presuming that astaxanthin accumulation is continuous until complete snowmelt due to an active metabolism; alternatively, the amount of chlorophyll could have decreased. This corresponds to a three-fold increase in α-tocopherol (this study), a major antioxidant of the chloroplasts. Similar α-tocopherol to chl-*a* ratios were observed in the ʻred phaseʼ of two algal strains isolated from Arctic snow and kept in conditions of low nitrogen and high irradiation (CCCryo 006-99 and 101-99/R2; Leya *et al*., 2009). Increased accumulation of α-tocopherol during spore maturation was reported for field samples of *C*. *nivalis* from the Alps (Remias *et al*., 2010) and in the stationary-growth phase of strains in a screening study by Mudimu *et al*. (2017). The harsher environmental conditions later in the season are in good agreement with the doubled amount of 13Z astaxanthin, which provides advanced protection from UV in relation to all-*trans*-astaxanthin (Supplementary fig. S22). Notably, astaxanthin as a bio-indicator was found in the entire profile of a sediment core (32 cm) taken from the deepest point of Gossenkӧlle Lake, representing the last 800 years (Kamenik *et al*., 2000). This may be associated not only with zooplankton grazing on algae containing astaxanthin (Tartarotti *et al*., 2017), but also with the presence of *Chlainomonas* sp. over time in the lake.

### Fatty acid composition

Snow-algal strains are reported as suitable candidates for biotechnological applications (Hulatt *et al*., 2017). In this study, *Chlainomonas* sp. had a high proportion of PUFAs in the total FA pool. High levels of PUFAs is quite common for aplanozygotes of snow-inhabiting members of the *Chloromonas* clade (Řezanka *et al*., 2008, 2014). α-Linolenic acid has been found as the dominant unsaturated FA of *Chlainomonas* sp. in other snow algae in this clade (Procházková *et al*., 2018). On the other hand, stearidonic and linoleic acids were twice as abundant as is typical for all 22 *Chloromonas* species (mean 7.6% and 6.4%, respectively) screened by Lang *et al*. (2011). Surprisingly, the FA profile of *Chlamydomonas nivalis* from the Austrian Alps was more similar to *Chlainomonas* sp. (e.g. in the dominance of α-linolenic acid) than to arctic field samples associated with the *Chlamydomonas nivalis* clade (oleic acid dominated >45% in sample 9/10 1b of Spijkerman *et al*., 2012). This suggests that the stage of spore maturation and differences in abiotic habitat parameters connected with latitude (e.g. irradiance, nutrients, water availability) may play a role in FA profiles in species belonging to the same phylogenetic clade (see fig. 4 in Leya *et al*., 2004). The culture maturation stage was also critical for the FA composition in Zygnematophycean green algae, where upon pre-akinete formation two unsaturated FAs in particular (oleic acid and linoleic acid) increased drastically (Pichrtová *et al*., 2016).

In summary, *Chlainomonas* sp. regularly causes red snow on lake-ice cover in the High Tatras and the Austrian Alps, as reported earlier by Remias *et al*. (2016). The differences in ecophysiology and morphology between these two populations illustrate well the influence of harsher conditions, prolonged time for spore maturation and population development during the later part of the growing season. The environmental conditions of the two high-alpine habitats seem to be similar. Several cell morphotypes corresponding to life-cycle stages (biflagellates, flagellates with collar-like papillae, quadriflagellates, mature spores) were found and quadriflagellate swarmers were suggested to be prolonged planozygotes. We hypothesize a route of lake-surface colonization by an inoculum originating from sediments, which first incubates in several slush layers prior to the appearance of the visible red colouration at the lake surface. Population patchiness along the spatial transect on the lake-ice cover was shown, no cell division was found, and the SWC varied from slush to nearly completely melted snow. Mating and cell divisions are thus presumed to take place in deeper slush layers and earlier in the season, where the SWC is most likely lower. Both *Chlainomonas* sp. and *C*. *nivalis* exhibited similar photosynthetic rates during high irradiance, suggesting that these snow algae are well adapted to live in open sites, although they have shown differences in their sensitivity to temperature and in the ultrastructural organization of plastids in mature spores (Remias *et al*., 2016). Both genera share low levels of primary pigments and a high contribution of non-polar astaxanthin esters. *Chlamydomonas nivalis* had a much lower PUFA content, partly due to the younger spore stages found during the sampling.

Remaining uncertainties about the life cycle of *Chlainomonas* sp. could be resolved by establishing a strain, which likely requires culturable, vegetative states occurring in deep slush layers early in the season. Polyphasic research at other, similar localities (alpine lakes, flat glaciers) with red-snow blooms is needed to assess the cosmopolitan distribution of this species, the morphology of dispersal cells, and the route(s) of colonization of distant locations. Analysis of multiple molecular markers of the other *Chlainomonas* cryoflora taxa would help to determine the exact taxonomic position of each species within the clade.

## Supplementary Material

Supplementary_material.docx

## Supplemental information

The following supplementary material is accessible via the Supplementary Content tab on the article’s online page at https://doi:10.1080/09670262.2018.1426789

**Supplementary text**. Climatic conditions

**Supplementary table S1**. List of primers used for amplification of 18S rDNA, ITS1 rDNA, ITS2 rDNA (ITS) and *rbc*L markers.

**Supplementary table S2**. List of taxa and GenBank accession numbers of nuclear-encoded 18S ribosomal DNA (rDNA) genes, nuclear rDNA internal transcribed spacer 1 (ITS1) and spacer 2 (ITS2) regions, and the large subunit of RuBisCO (*rbc*L) genes.

**Supplementary table S3**. List of *Chlainomonas* species causing red snow.

**Supplementary table S4**. Relative content of pigments and α-tocopherol in ratios to chlorophyll-*a* (=1) in field samples of *Chlainomonas* sp. from the High Tatras (sample LP03) and the Austrian Alps (sample DL06), determined by HPLC.

**Supplementary figs S1**–**S4**. Overview of the sampling site of the snow alga *Chlainomonas* sp. at Gossenkӧlle Lake (Tyrolean Alps, Austria).

**Supplementary figs S5–S12**. LM micrographs of *Chlainomonas* sp. showing cells from the snow on the ice cover of Gossenkӧlle Lake after harvest and after several months in lab conditions.

**Supplementary fig. S13**. A swarmer related to *Chlainomonas rubra* found in red snowfields nearby to Gossenkӧlle lake.

**Supplementary figs S14–S17**. TEM and SEM micrographs of *Chlainomonas* sp. from the snow on the ice cover of Gossenkӧlle Lake.

**Supplementary figs S18, S19**. Spatial distribution of *Chlainomonas* sp. population and snow water content on the southern-northern transect on the ice cover of Gossenkӧlle Lake.

**Supplementary figs S20, S21**. HPL-chromatogram of *Chlainomonas* sp. at 480 nm.

**Supplementary fig. S22**. Spectral absorbance of all-*trans*-astaxanthin and of the isomer 13Z astaxanthin.

**Supplementary figs S23, S24**. Prevailing climatic conditions close to habitats of *Chlainomonas* sp.

**Supplementary figs S25, S26**. Daily mean air temperature in period when the bloom of *Chlainomonas* sp. is expected (May and June 2016) at ice covers of Ľadové Lake and Gossenkӧlle Lake.

**Supplementary figs S27, S28**. Daily course of photosynthetically active radiation (PAR) in a few contrasting days during May–June.

**Supplementary fig. S29**. Daily course of PAR (photosynthetic active radiation; µmol photons m^–2^ s^–1^) during May–June 2016 in the proximity of Gossenkӧlle Lake.
