# Supplementary material for: Ecophysiological and morphological comparison of two populations of Chlainomonas sp. (Chlorophyta) causing red snow on ice-covered lakes in the High Tatras and Austrian Alps
Source: Eur J Phycol. 2018 Apr 4;53(2):230–43. doi: 10.1080/09670262.2018.1426789 (PMC5940174; doi:10.1080/09670262.2018.1426789)
Supplement: Supplementary_material.docx [file TEJP_A_1426789_SM8075.docx]

**Supplemental information**

**Climatic conditions**

The values for the High Tatras originated from an automatic weather station located on the shore of the Ľadové Lake: global radiation (GR, in 2001, unpublished data), precipitation (in 2001, Křeček *et al.*, 2006), air temperature data (in 2016, provided by Stredisko lavínovej prevencie Horskej záchrannej služby, Slovakia). Air temperature, irradiance, and precipitation data for the Tyrolean Alps during 2016 were kindly provided by the company Tiwag, Innsbruck (weather station close to Längentaler Stausee, N 47.207149 E 11.005895, 1914 m above sea level). Since this station is located in the same valley as Gossenkӧlle Lake but at an altitude 500 m lower, monthly mean air temperatures measured at the meteorological station were extrapolated taking the dry adiabatic vertical temperature gradient into account (–0.65°C per increase of 100 m) to estimate air temperature at the elevation of the lake. Photosynthetically active radiation (PAR, in units of µmol photons m^–2^ s^–1^) was calculated from GR (in units of W m^–2^): multiplied by 50% for the fraction of light in global radiation (output in J m^–2^ s^–1^) and then multiplied by 4.6 (conversion factor for joule to micromole). *In situ* PAR for Gossenkӧlle Lake (May 28–30, 2017) was monitored using a data logger with built-in sensors and equipped with radiation shields (Minikin QT, EMS Brno, Czech Republic), which was installed on the rock surface close to the lake ice cover.

**Supplemental tables**

**Supplementary Table S1**. List of primers used for amplification of 18S rDNA, ITS1 rDNA, ITS2 rDNA (ITS) and *rbc*L markers; F **−** forward; R **−** reverse.

| Primer | Marker | Direction | Sequence | Reference |  |
| --- | --- | --- | --- | --- | --- |
| P2 | 18S | F | CTGGTTGATTCTGCCAGT | Gargas & DePriest (1996) | |
| P4 | 18S | R | TGATCCTTCYGCAGGTTCAC | Moon-van der Staay *et al.* (2000) | |
| TW81 | ITS | F | GGGATCCGTTTCCGTAGGTGAACCTGC | Goff *et al.* (1994) | |
| AB28 | ITS | R | GGGATCCATATGCTTAAGTTCAGCGGGT | Goff *et al.* (1994) | |
| AL1500af | ITS | F | GCGCGCTACACTGATGC | Helms *et al.* (2001) | |
| LR3 | ITS | R | GGTCCGTGTTTCAAGACGG | Vilgalys & Hester (1990) | |
| ITS5 | ITS | F | GGAAGTAAAAGTCGTAACAAGG | White *et al.* (1990) | |
| ITS4 | ITS | R | TCCTCCGCTTATTGATATGC | White *et al.* (1990) | |
| *rbc*L1F | *rbc*L | F | GCTGGTGTTAAAGATTATCG | Hoham *et al.* (2002) | |
| *rbc*L7R | *rbc*L | R | AAATAAATACCACGGCTACG | Hoham *et al.* (2002) |  |

**Supplementary Table S2**. List of taxa and GenBank accession numbers of nuclear-encoded 18S ribosomal DNA (rDNA) genes, nuclear rDNA internal transcribed spacer 1 (ITS1) and spacer 2 (ITS2) regions, and the large subunit of RuBisCO (*rbc*L) genes.

| Taxon | Specimen | Accession number | | | |
| --- | --- | --- | --- | --- | --- |
|  |  | 18S rDNA | ITS1 rDNA | ITS2 rDNA | *rbc*L |
| *Chlainomonas* sp. | LP03 | MF803745 | MF803746 | MF803746 | MF803747 |
| *Chlainomonas* sp. | DL06 | MF803743 | MF803743 | MF803743 | MF803744 |
| *Chlamydomonas nivalis* | DL07 | MF803748 |  | MF803749 |  |
|  |  |  |  |  |  |

**Supplementary Table S3**. List of *Chlainomonas* species causing red snow: cell sizes of different life cycle stages in µm; flagel. = flagellates.

| Taxon | Location | life cycle stages | | | | | Reference |
| --- | --- | --- | --- | --- | --- | --- | --- |
|  |  | uncollar  quadriflagel. | collar quadriflagel. | biflagel. | immotile stages | mature spores |  |
| *Chlainomonas* sp. | Ľadové Lake | 24–65 ×  24–59 | 19.1 × 19 | 19.4 × 8.8 | 65 (85) ×  59 (83) | | this study |
| *Chlainomonas* sp. | Gossenkӧlle Lake | 20.4–46 × 19–45.7 | 23–28 ×  19.5–25.4 |  | 46 × 45.7 | 20–40 | this study |
| *Chlainomonas rubra* | Austria | 42–55 ×  34–50(70) |  |  |  |  | Ettl (1968) |
| *Chlainomonas rubra* | North America | 23–42(60) × 30–55(90) |  | 17–25 × 6–10 |  | 30–63 × 25–48 | Hoham (1974*a*,  1974*b*) |
| *Chlainomonas kolii* | New Zealand |  | (15)20–30 × (10)15–22 | 18 × 10 |  |  | Novis (2002*a*) |
| *Chlainomonas kolii* | North America |  | 28–38 ×  14–22 |  |  | 13–16 × 14–19 | Hardy & Curl (1968),  Hoham (1974*a*) |

**Supplementary Table S4**. Relative content of pigments and α-tocopherol in ratios to chlorophyll a (=1) in field samples of *Chlainomonas* sp. from the High Tatras (sample LP03) and the Austrian Alps (sample DL06), determined by HPLC. Note that in LP03 the de-epoxydised xanthophyll-cycle pigment zea is present, and it has also a higher content of α-tocopherol. The total amount of astaxanthin (ast-tot and asI-tot thereof) is 26.178 to 17.594 in favour to LP03. Abbreviations: neo, neoxanthin; vio, violaxanthin; ant, antheraxanthin; lut, lutein; zea, zeaxanthin; ech, echinenone; chl *b*, chlorophyll b; ast-tot = astaxanthin all-*trans* plus astaxanthin esters; asi-tot, total amount of astaxanthin *cis*-isomers (mainly 13*Z*); α-toc, α-tocopherol; n.d., not detected.

|  | Neo | Vio | Ant | Lut | Zea | Ech | Chl *b* | Ast-tot | AsI-tot | α-toc |
| --- | --- | --- | --- | --- | --- | --- | --- | --- | --- | --- |
| LP03 | 0.05 | 0.177 | 0.00 | 0.252 | 0.026 | 0.00 | 0.444 | 14.531 | 11.648 | 0.397 |
| DL06 | 0.053 | 0.018 | 0.00 | 0.359 | n. d. | 0.00 | 0.583 | 11.981 | 5.613 | 0.122 |

**Supplemental figures**

**
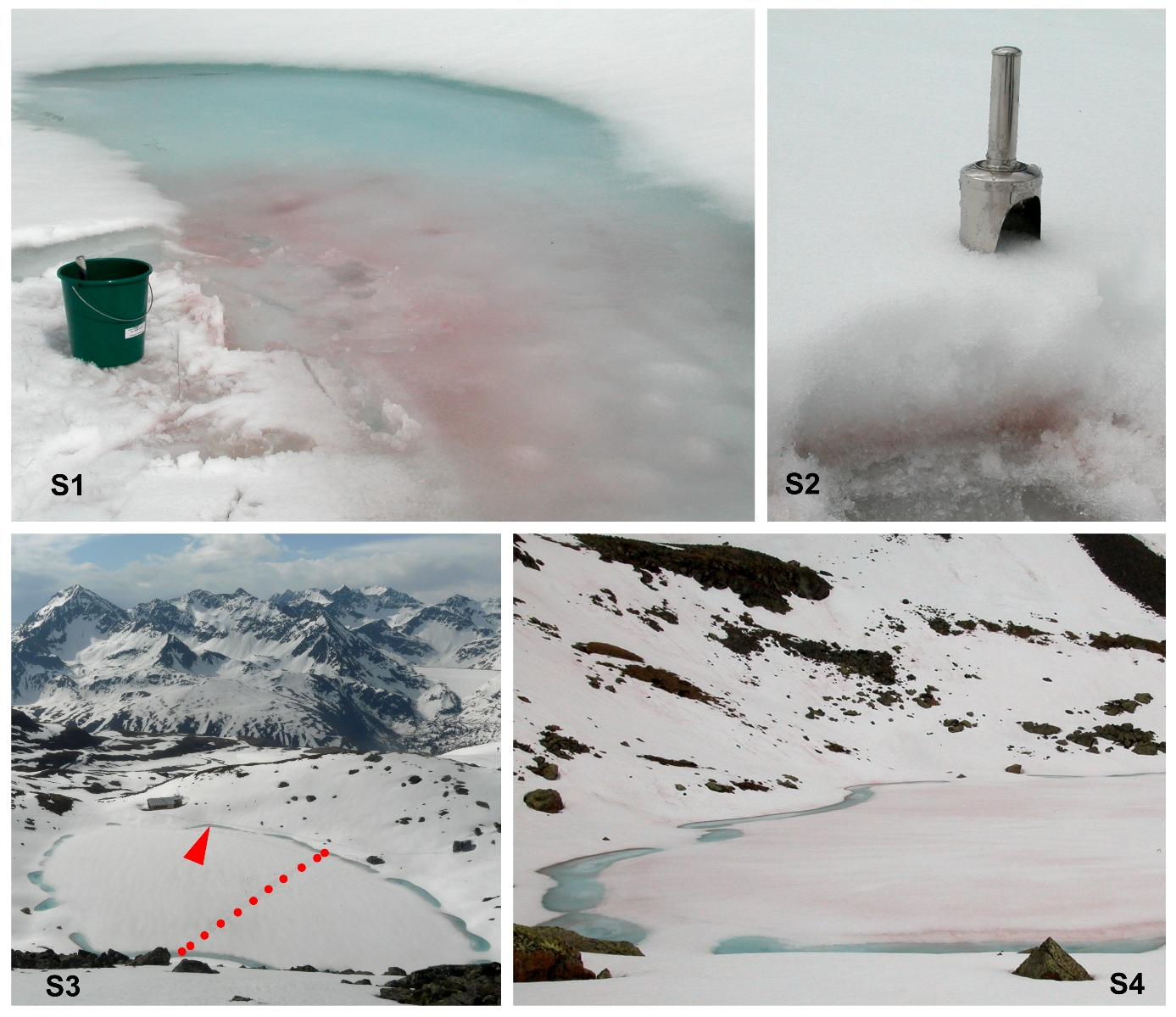
**

**Supplementary Figs S1**–**S4**. Overview of the sampling site of the snow alga *Chlainomonas* sp. at Gossenkӧlle Lake (Tyrolean Alps, Austria). **Fig. S1**. During harvest, the snow colouration was visible only close to the lake margin, where melting of the underlying ice was advanced (late May 2016), detail view of red snow after harvest (sample DL06). **Fig. S2**. The majority of the lake surface was still covered by white snow and red horizontal patches of snow were hidden several cm below the snow surface, close to the interface with the ice cover. **Fig. S3**. Population densities and snow water contents were investigated in a 109 m long southern-northern transect on the ice cover of the lake. The main sampling location (DL06) was close to the southern shore (red arrowhead). **Fig. S4**. Red spots appeared one week later across the entire lake snow surface, most likely due to ongoing melting processes (early June 2016).

**
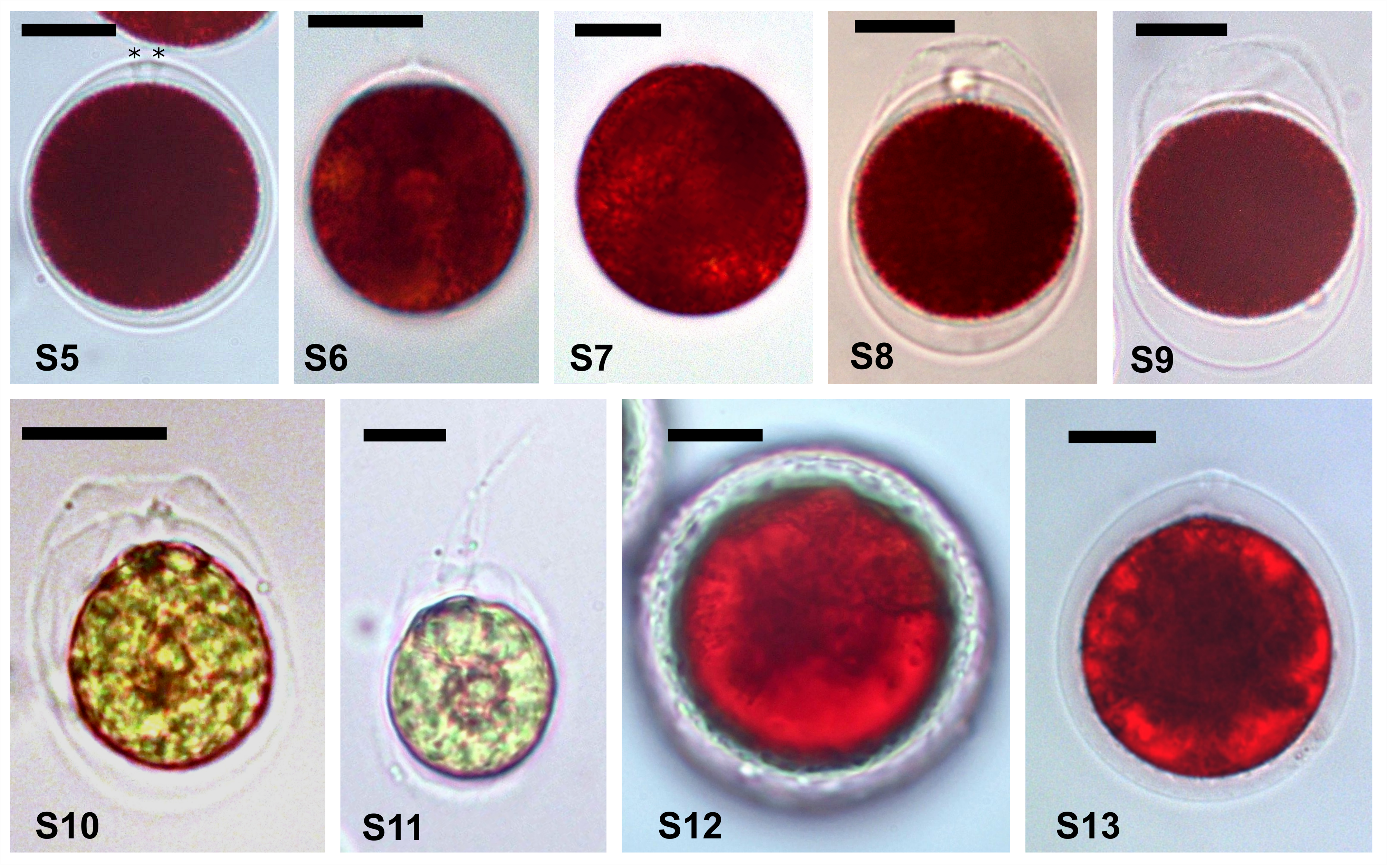
 Supplementary Figs S5–S12**. LM micrographs of *Chlainomonas* sp. showing cells from the snow on the ice cover of Gossenkӧlle Lake after harvest (Figs S5–S9, living cells; Figs S10–S11, fixed material with acid Lugol´s solution) and after several months at lab conditions (Fig. S12). **Figs** **S5–S11**. Morphological variability of swarmers. **Fig. S5**. Most abundant swarmers possessed papilla and pseudo-papilla (presumably a zygote), two pairs of flagella groves of a swarmer marked with asterisk. **Figs S6, S7**. Swarmers with equally thin cell wall and more or less apparent papilla. **Figs S8–S11**. Flagellates with collar papilla. **Fig. S9**. A cell in a process of losing the collar. **Fig. S12**. Mature spores with thick secondary cell wall. **Fig. S13**. A swarmer related to *Chlainomonas rubra* found in red snowfields nearby to Gossenkӧlle Lake. Note the secondary cell wall equally distant from protoplast and small projections of the inner cell wall. Scale bar = 10 µm.

**
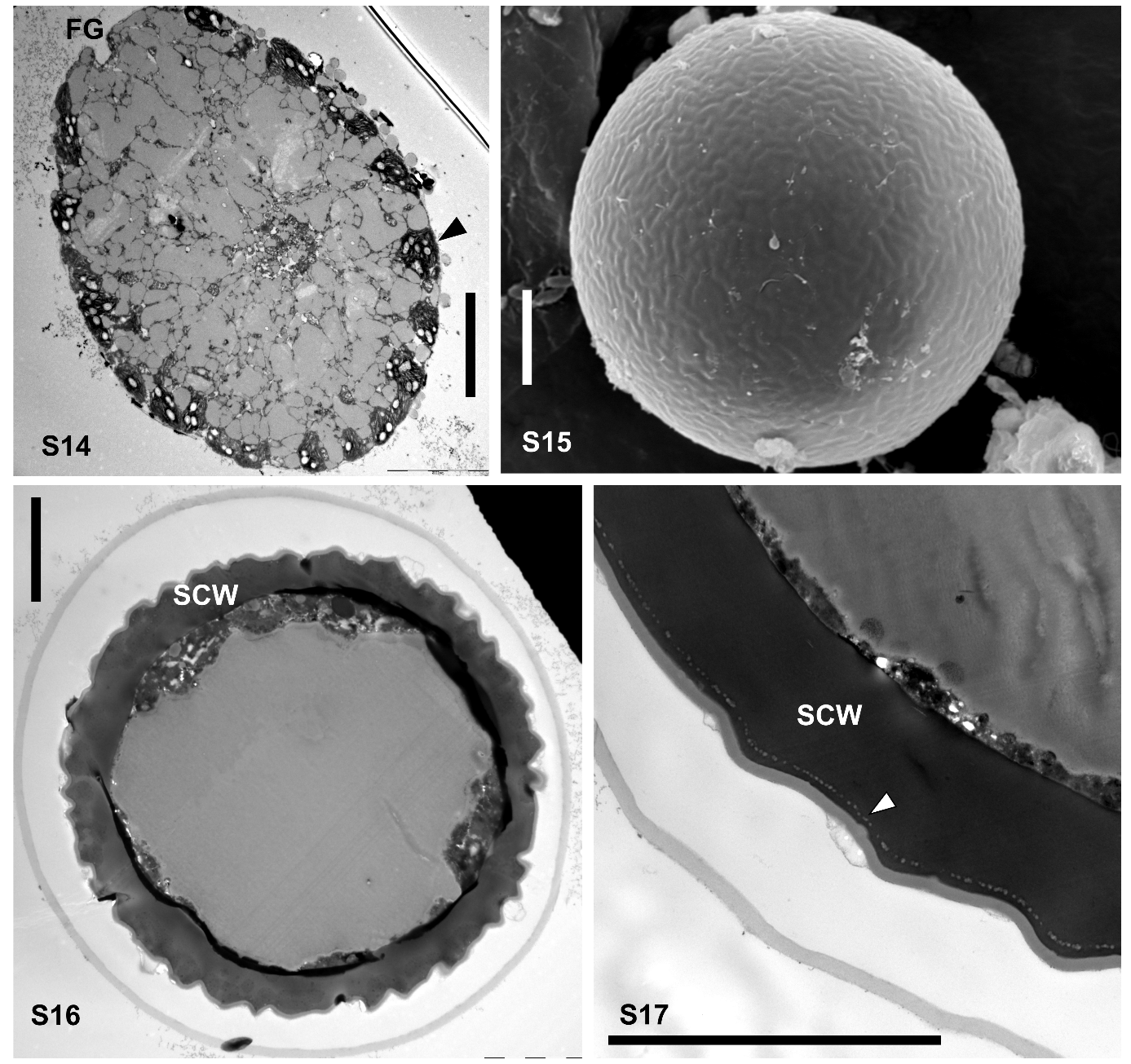
 Supplementary Figs S14–S17**. TEM and SEM micrographs of *Chlainomonas* sp. from the snow on the ice cover of Gossenkӧlle Lake. **Fig. S14**. Swarmer with equally thin cell wall, parietal plastids (black arrow) and flagellar groove (FG). **Figs S15–S17**. Mature spore after several months at lab conditions, note undulating fine surface structures (corresponding to Fig. S12). Section of a trilaminar sheath (white arrow) and fully developed secondary cell wall (SCW), surrounded by extracellular matrix. Scale bar = 5 µm.

**
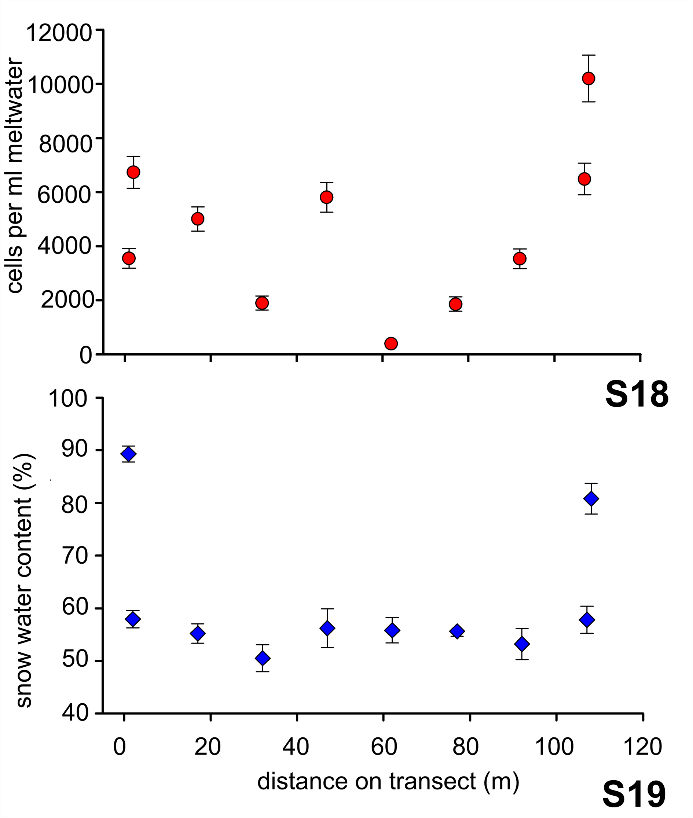
**

**Supplementary Figs S18, S19**. Spatial distribution of *Chlainomonas* sp. population (red circles) and snow water content (SWC, blue boxes) on the southern-northern transect on the ice cover of Gossenkӧlle Lake. Each point of population density and SWC represents the mean of two and three measurements, respectively. Standard deviation is indicated.

**
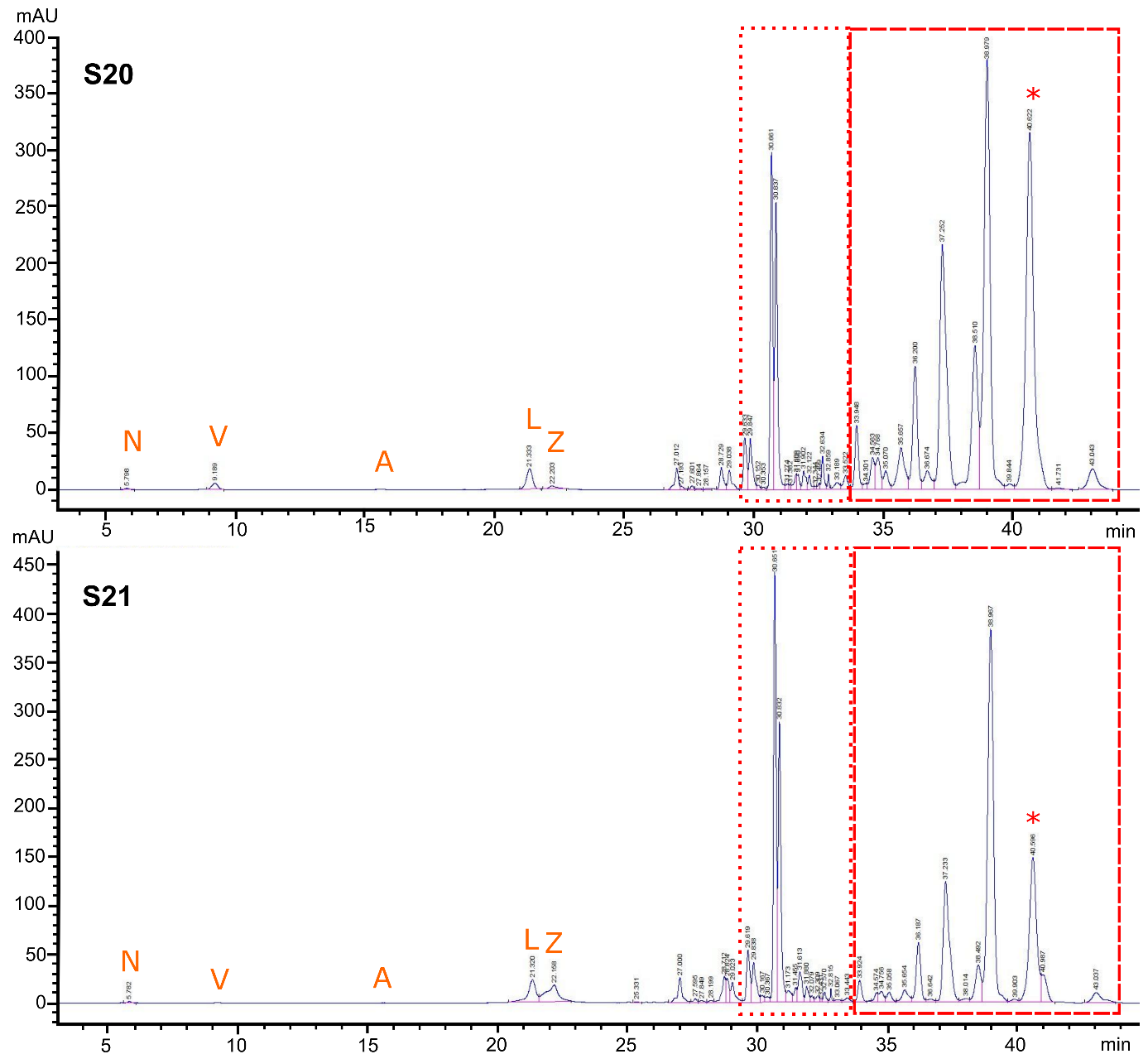
 Supplementary Figs S20, S21**. HPL-chromatogram of *Chlainomonas* sp. at 480 nm. **Fig. S20**. Sample harvested in the High Tatras. **Fig. S21**. Sample harvested in the Tyrol Alps. Two groups of peaks are indicated: Astaxanthin-monoesters (red dotted line), astaxanthin-diesters (red dashed line). Abbreviations: N, neoxanthin; V, violaxanthin; A, antheraxanthin; L, lutein; Z, zeaxanthin; 13*Z* isomers astaxanthin indicated by asterisk.

**
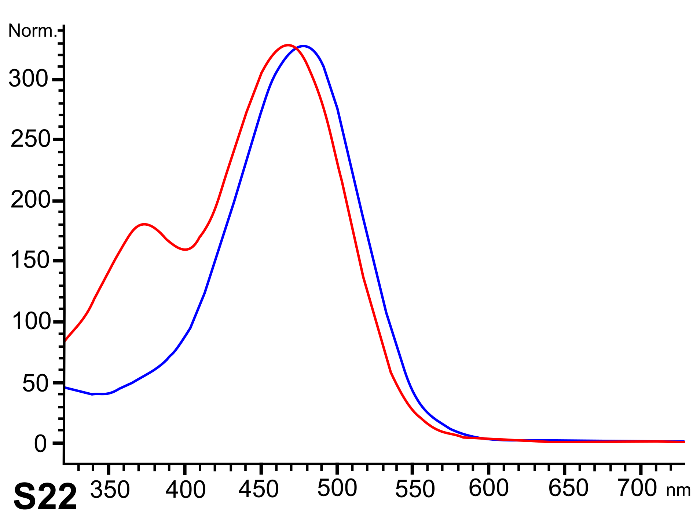
**

**Supplementary Fig. S22**. Spectral absorbance of all-*trans*-astaxanthin (blue line, maximum around 478 nm) and of the isomer 13Z astaxanthin with a similar maximum absorbance but providing additional UV protection (red line, shoulder at 371 nm). Peaks with these spectra were found in both *Chlainomonas* sp. samples.


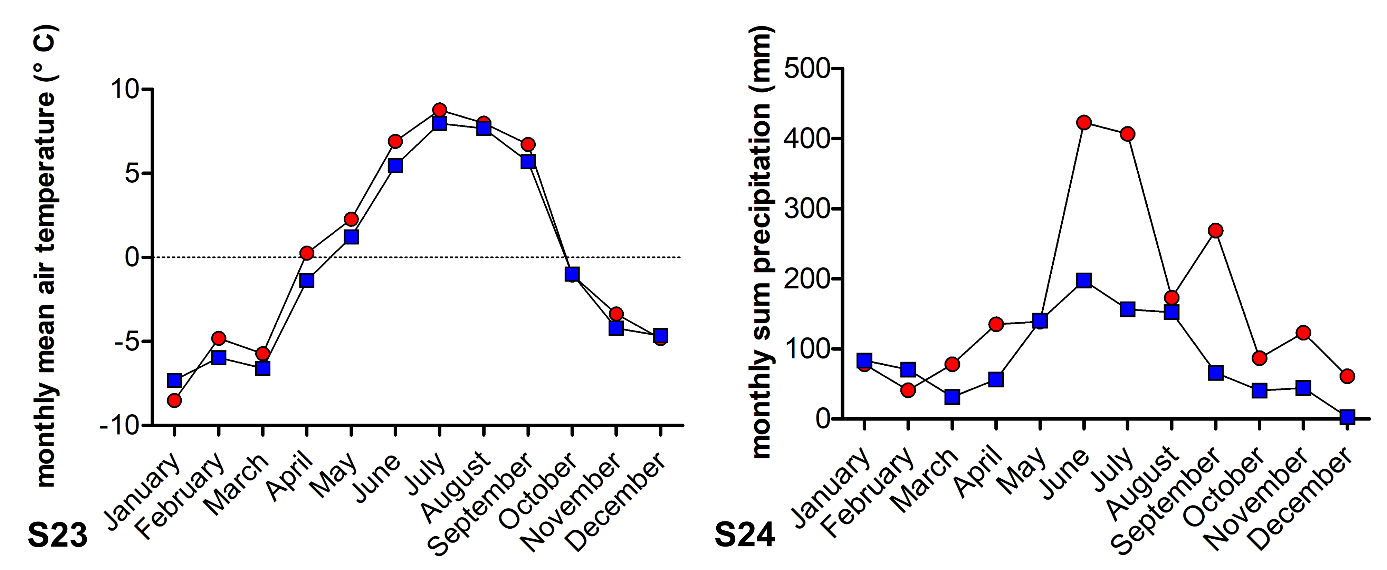


**Supplementary Figs S23, S24**. Prevailing climatic conditions close to habitats of *Chlainomonas* sp. (Ľadové Lake – circles, Gossenkӧlle Lake – boxes). **Fig. S23**. Monthly sum of precipitation (mm). **Fig. S24**. Monthly mean air temperature (°C).


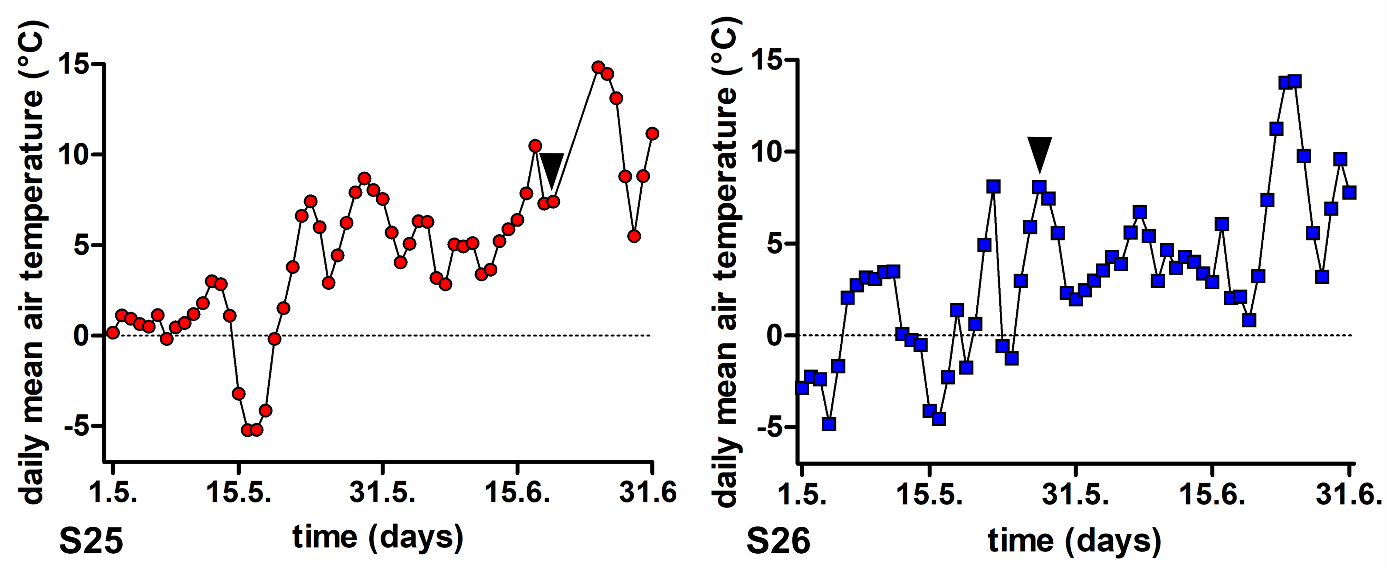


**Supplementary Figs S25, S26**. Daily mean air temperature in period when the bloom of *Chlainomonas* sp. is expected (May and June 2016) at ice covers of Ľadové Lake (red circles) and Gossenkӧlle Lake (blue squares). Black arrows indicate the date of red snow harvest in course of this study. Despite comparable prevailing air temperatures at both dates of sampling, there was a much longer period of time since the last freezing events at Ľadové Lake (one month) in comparison to Gossenkӧlle Lake (two days), thus indicating an advanced snowmelt and associated higher exposure of cells to the irradiance on the snow surface in the former.


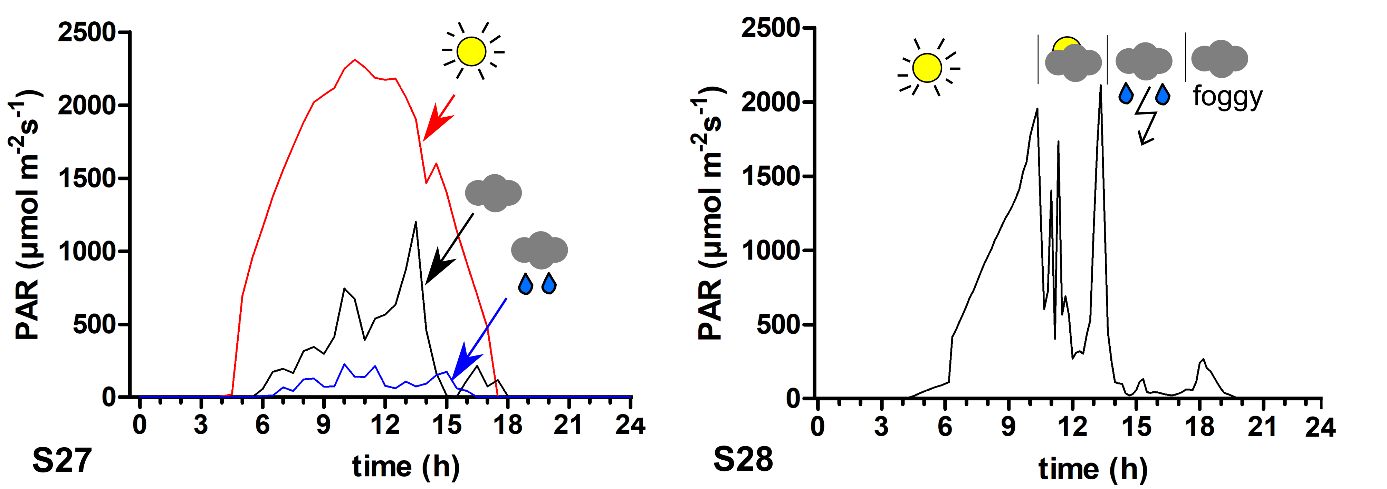


**Supplementary Figs S27, S28**. Daily course of photosynthetically active radiation (PAR) in a few contrasting days during May-June. **Fig. S27**. At Ľadové Lake: sunny (red line, 23. 5. 2001), cloudy (black line, 1. 6. 2001) and rainy day (blue line, 19. 6. 2001). **Fig. S28**. Changeable weather conditions during one day *in situ* at Gossenkӧlle Lake (30. 5. 2017) from sunny to cloudy, rainy and then foggy. Note the difference in timing of sunset at both lakes in studied period caused partly by actual weather conditions and partly by different topographic shading: Ľadové Lake is surrounded by mountain peaks except on the south and south-southwest and thus it receives less direct solar input. In contrast, Gossenkӧlle Lake is surrounded by mountain peaks only to the north, and is less affected by more-distant peaks to the northwest, which means that sunshine at this location continues until the late evening.


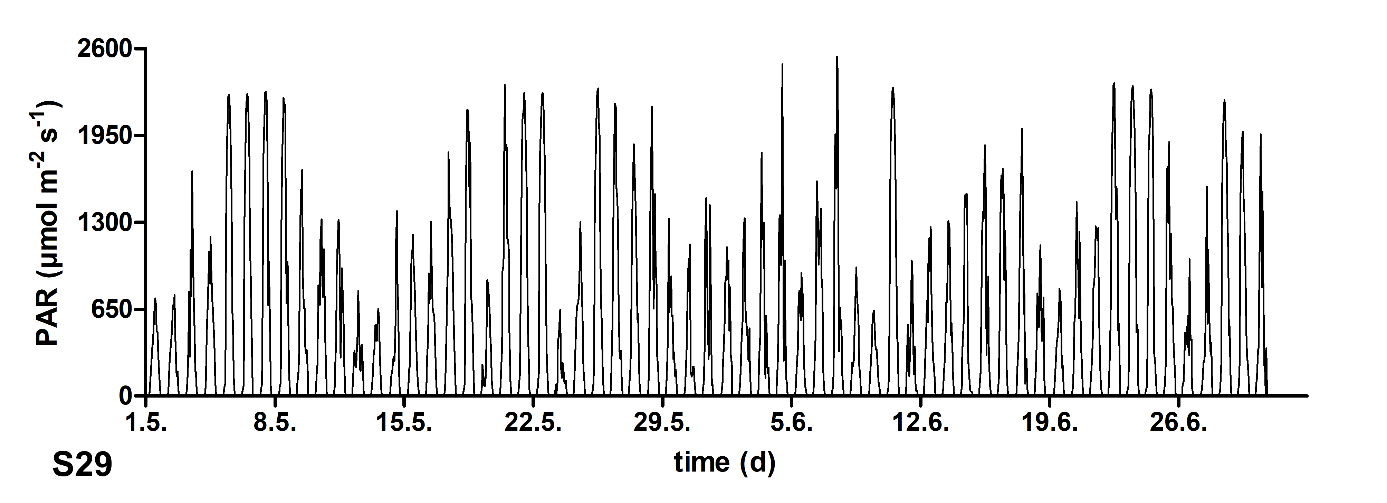


**Supplementary Fig. S29**. Daily course of PAR (photosynthetic active radiation; µmol photons m^–2^ s^–1^) during May-June 2016 in the proximity to Gossenkӧlle Lake.

**ADDITIONAL REFERENCES**

Gargas, A. & DePriest, P. T. (1996). A nomenclature for fungal PCR primers with examples from intron-containing SSU rDNA. *Mycologia*, **88(5)**: 745–748.

Goff, L.J., Moon, D.A. & Coleman, A.W. (1994). Molecular delineation of species and species relationships in the red algal agarophytes *Gracillariopsis* and *Gracilaria* (Gracilariales). *Journal of Phycology*, **30**: 521–537.

Helms, G., Friedl, T., Rambold, G. & Mayerhofer, H. (2001). Identification of photobionts from the lichen family Physciaceae using algal-specific ITS rDNA sequences. *Lichenologist*, **33**: 73–86.

Hoham, R.W., Bonome, T.A., Martin, C.W. & Leebens-Mach, J.H. (2002). A combined 18S rDNA and *rbc*L phylogenetic analysis of *Chloromonas* and *Chlamydomonas* (Chlorophyceae, Volvocales) emphasizing snow and other cold-temperature habitats. *Journal of Phycology*, **38**: 1051–1064.

Křeček, J., Turek, J., Ljungren, E., Stuchlík, E. & Šporka, F. (2006). Hydrological processes in small catchments of mountain headwater lakes: The Tatra Mountains. *Biologia*, **61**: S1–S10. https://doi.org/10.2478/s11756-006-0115-8

Moon-van der Staay, S.Y., van der Staay, G.W.M., Guillou, L. & Vaulot, D. (2000). Abundance and diversity of prymnesiophytes in the picoplankton community from the equatorial Pacific Ocean inferred from 18 rDNA sequences. *Limnology and Oceanography*, **45(1)**: 98–109.

Vilgalys, R. & Hester, M. (1990). Rapid genetic identification and mapping of enzymatically amplified ribosomal DANN from several *Cryptococcus* species. *Journal of Bacteriology*, **175**: 4238–4246.

White, T.J., Bruns, T., Lee, S.J.W.T. & Taylor, J.W. (1990). Amplification and direct sequencing of fungal ribosomam RNA genes for phylogenetics. *PCR protocols: a guide to methods and applications*, **18(1)**: 315–322.
